# The Validity of Stryd Leg Stiffness Against the Morin (2005) Sine-Wave Method: A Level-1 Assessment of Flat and Uphill Treadmill Running

**DOI:** 10.3390/s26103244

**Published:** 2026-05-20

**Authors:** Diego Jaén-Carrillo, Antonio Cartón-Llorente

**Affiliations:** 1 Department of Sport Science, University of Innsbruck, 6020 Innsbruck, Austria; diego.jaen@uibk.ac.at; 2 Faculty of Health Sciences, Universidad San Jorge, 50830 Zaragoza, Spain

**Keywords:** spring–mass model, trail running, validation, wearable technology

## Abstract

This study evaluated the validity of the leg stiffness metric provided by the Stryd running power meter against the Morin (2005) sine-wave spring–mass model. Twenty-three highly trained trail runners (11 women) completed a 12 min uphill time trial at +12% grade and one hour of submaximal level running. Leg stiffness was calculated from contact time, flight time, running speed, and leg length using Morin’s method, and compared with Stryd values. Agreement was assessed following the Dhahbi and Chamari Level-1 analytical framework, including intraclass correlation coefficient (ICC_2,1_), Bland–Altman analysis, mean absolute percentage error (MAPE), and paired *t*-tests. Stryd and Morin estimates showed excellent agreement in both conditions: uphill running: ICC_2,1_ = 0.96 (95%CI: 0.91–0.98), bias = −0.02 kN·m^−1^, limits of agreement (LoAs) = [−0.61, 0.58] kN·m^−1^, MAPE = 2.5% (*p* = 0.803); and level running: ICC_2,1_ = 0.97 (95%CI: 0.93–0.99), bias = −0.04 kN·m^−1^, LoAs = [−0.62, 0.54] kN·m^−1^, MAPE = 2.6% (*p* = 0.505). The Stryd sensor provides valid leg stiffness estimates in highly trained trail runners on both level and inclined terrain. The negligible systematic bias and narrow limits of agreement support the use of Stryd for leg stiffness monitoring in field and laboratory settings.

## 1. Introduction

The Stryd running foot pod (Stryd Inc., Boulder, CO, USA) has become one of the most widely adopted wearable devices in running and trail running research. An expanding body of the peer-reviewed literature has used Stryd-derived data to investigate running power output, biomechanics, and performance across laboratory and field settings [1,2,3,4]. This proliferation creates a scientific obligation: each metric must be independently validated against established references before use with confidence in research or applied practice [5].

Several Stryd metrics have been formally validated. Stride kinematics such as cadence, stride length, and contact time have demonstrated strong concurrent validity against optical and force-plate references across a range of running velocities [2,6]. Running power has been evaluated against metabolic cost and mechanical power with good linear agreement [1]. Reliability under inclined and trail conditions has been confirmed for speed, cadence, and contact time [7], and Stryd biomechanical parameters have been shown to predict performance in trained runners [3]. In contrast, leg spring stiffness (k_leg_) has received comparatively little analytical attention. The spring–mass model conceptualizes the runner as a mass oscillating on a linear spring that compresses and recoils during ground contact [8,9], with k_leg_ directly linked to running economy and stride regulation. To date, only Imbach et al. [1] have compared Stryd k_leg_ against a laboratory reference (i.e., force platform), finding acceptable agreement in a sample of six recreational runners completing flat treadmill running. No study has examined the validity of Stryd k_leg_ on inclined terrain, even though uphill running is a defining feature of trail running and mountain racing, contexts in which Stryd is increasingly deployed.

The Dhahbi & Chamari [5] standardization framework for wearable assessment technologies prescribes Level-1 analytical validation (i.e., direct comparison against an established reference, with ICC > 0.7, Bland–Altman analysis, and MAPE as primary metrics) as the mandatory first step before applied adoption. Morin et al. [10] validated a sine-wave method to estimate kleg from body mass, velocity, leg length, contact time, and flight time, reporting biases of 0.12–6.93% against force platforms. The purpose of this study is therefore to conduct a Level-1 analytical validation of Stryd kleg against the sine-wave method [10] under flat (0%) and inclined (+12%) treadmill running conditions.

## 2. Materials and Methods

Twenty-three highly trained trail runners (12 males, 11 females; body mass 67.7 ± 9.4 kg; height: 1.73 ± 0.1; leg length: 0.92 ± 0.1) participated in the parent study (Jaén-Carrillo et al.) [11], from which this work constitutes a secondary analysis. Briefly, participants were required to have ≥2 years of trail running experience, run ≥50 km per week with ≥800 m of elevation gain, and be free from musculoskeletal injuries. All tests were conducted on a motorized treadmill under controlled environmental conditions (21–24 °C, 45–55% relative humidity). The study was approved by the Ethics Committee of the University of Innsbruck (no. 109/2024) and conformed to the Declaration of Helsinki; all participants provided written informed consent. Full protocol details are available in the parent publication [11]. Data were collected under two treadmill conditions (hp cosmos, h/p/cosmos sports & medical GmbH, Nussdorf-Traunstein, Germany) separated by 48 h: (i) uphill running: a 12 min time trial at +12% gradient performed in fresh state (mean speed 2.59 ± 0.34 m·s^−1^); and (ii) level running: the first hour of a 180 min submaximal treadmill run at 85% of the speed corresponding to the lactate threshold +0.5 mmol·L^−1^ at 0% gradient (mean speed 3.10 ± 0.41 m·s^−1^). All data represent condition-level participant means recorded by the Stryd foot pod. The study conformed to the Declaration of Helsinki; all participants provided written informed consent.

The Stryd foot pod, clipped to the laces of the running shoes, provided contact time (CT, ms), flight time (FT, ms), running speed (v, m·s^−1^), and leg stiffness (kN·m^−1^). Leg spring stiffness (k_leg_) is defined as the ratio of the peak ground reaction force to the maximum leg spring compression during stance, and characterizes the global mechanical behavior of the lower limb as a spring during the contact phase of running [8,9]. Higher k_leg_ is generally associated with better running economy, stiffer elastic energy storage and return, and a more efficient stride [7,8]. Leg stiffness was estimated following Morin et al.’s sine-wave method [10]. Modeled peak ground reaction force:

F̂_max_ = m·g·(π/2)·(t_f_/t_c_ + 1) (1)

where m is body mass (kg), g = 9.81 m·s^−2^, t_f_ = flight time (s), and t_c_ = contact time (s). Vertical center-of-mass displacement:

Δŷ_c_ = F̂_max_·t_c_^2^/(m·π^2^) − g·t_c_^2^/8 (2)

Peak leg spring compression:

ΔL̂ = L − √(L^2^ − (v·t_c_/2)^2^) + Δŷ_c_ (3)

Leg stiffness:

k_leg_ = F̂_max_/ΔL̂ (4)

Leg length was estimated as L = 0.53·h using sex-based mean heights (males: 1.78 m, L = 0.943 m; females: 1.65 m, L = 0.875 m). Morin et al. [10] reported only a 1.94 ± 1.51% error using this approach versus directly measured leg length. It should be noted that highly trained trail runners may present body proportions that differ slightly from general population norms; however, given the low error reported by Morin et al. [10] and the negligible mean biases observed in both conditions of the present study, this approach is considered acceptable for a Level-1 assessment. Future studies should prioritize direct measurement of leg length to eliminate this potential source of individual-level variability.

The statistical analysis followed Dhahbi & Chamari [5]. Level-1 validation included: (i) ICC(2,1) with absolute agreement (two-way random effects, single measures), threshold ICC > 0.7; (ii) Pearson r and R^2^; (iii) Bland–Altman analysis (mean bias and 95% LoA); (iv) MAPE; and (v) paired *t*-test (α = 0.05). ICC 95% CIs were computed via the F-distribution method [12]. To examine whether the LoA remained stable across the measurement range (heteroscedasticity), a Pearson correlation was computed between the absolute bias values (|Stryd − Morin|) and the mean of the two methods for each participant; non-significant correlations (both *p* > 0.05) confirmed the absence of proportional bias. All analyses used Python 3.10 (SciPy 1.11).

## 3. Results

The uphill running condition (+12%) yielded shorter flight times and longer contact times than level running, reflecting the mechanical response to uphill running. Mean k_leg_ was similar across conditions and methods (Table 1).

### Level-1 Analytical Validation

**Figure 1:**
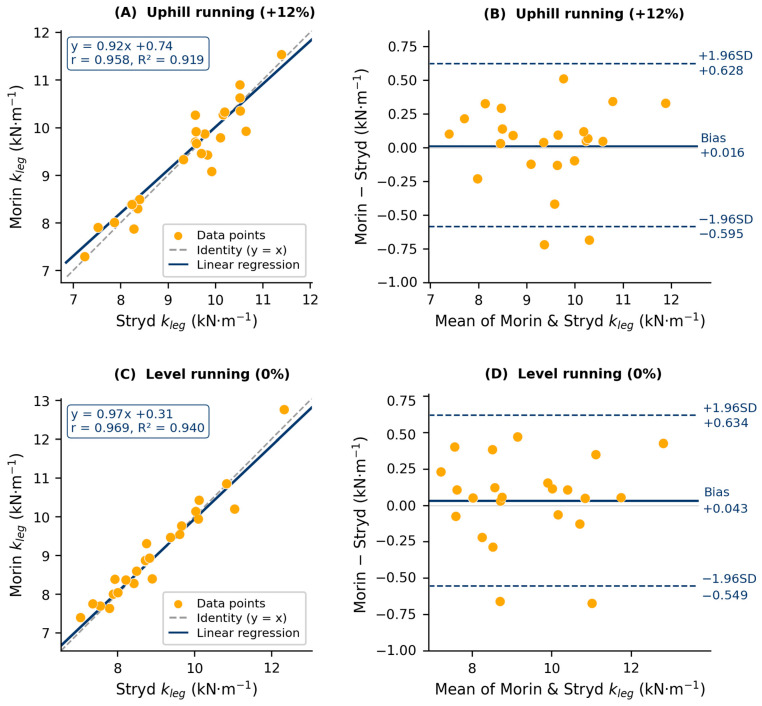
Scatter plots and Bland–Altman plots for the comparison between Stryd-reported and Morin (2005) sine-wave estimates of leg spring stiffness (kleg) under two treadmill conditions. Upper row (panels (**A**,**B**)): uphill running at +12% gradient. Lower row (panels (**C**,**D**)): level running at 0% gradient. Panels (**A**,**C**) (left): individual data points with the identity line (y = x, dashed) and the linear regression line (solid). Panels (**B**,**D**) (right): Bland–Altman plots displaying the mean difference (Stryd − Morin; central solid line) and the 95% limits of agreement (±1.96 SD; outer dashed lines), with numerical values annotated on the right side of each panel.

All Level-1 criteria were met and substantially exceeded in both conditions (Table 2). For TT-Control (+12% grade), ICC(2,1) values of 0.959 and 0.970 for uphill and level running, respectively, are classified as ‘excellent’ (>0.90) according to Koo and Li [12]. The paired *t*-test revealed no statistically significant difference between Stryd and Morin estimates in either condition (uphill: *p* = 0.803; level: *p* = 0.505). Bland–Altman analysis confirmed near-zero systematic bias in both conditions, both well within acceptable limits. Full statistics are reported in Table 2. At the individual level, the maximum absolute bias observed was 0.62 kN·m^−1^ (uphill) and 0.63 kN·m^−1^ (level), consistent with the outer LoA boundaries, while the minimum absolute bias was <0.01 kN·m^−1^ in both conditions. The heteroscedasticity analysis confirmed no significant correlation between the absolute bias and the mean of the two methods in either condition (both *p* > 0.05), indicating that agreement was uniform across the range of k_leg_ values observed. Scatter plots and Bland–Altman plots are presented in Figure 1.

## 4. Discussion

Applying the Level-1 framework of Dhahbi & Chamari [5], Stryd k_leg_ satisfies all prescribed validation criteria with large margins. ICC(2,1) values of 0.959 and 0.970 far exceeded the threshold of 0.70, falling within the ‘excellent’ range (>0.90) [12]. MAPE values below 3% and near-zero Bland–Altman biases (<0.05 kN·m^−1^) are comparable to the reference-model biases of 0.12–6.93% reported by Morin et al. [10] against force platforms. These findings considerably extend the sole prior comparison by Imbach et al. [1], limited to six recreational runners on flat terrain, by confirming validity in a larger sample and under a +12% gradient. The ICC values obtained here are consistent with the excellent concurrent validity previously reported for other Stryd stride kinematic parameters (cadence, stride length, contact time) against optical references [2,6], and extend this evidence base to kleg. Moreover, within the Dhahbi & Chamari [5] framework, all three primary criteria (ICC > 0.70, MAPE < 5%, and absence of significant systematic bias) were simultaneously satisfied, reinforcing the robustness of the Level-1 verdict.

The validity of Stryd k_leg_ on inclined terrain is particularly relevant given the growing application of Stryd in trail running and mountain racing [4,7]. The +12% gradient condition yielded negligible mean bias (−0.016 kN·m^−1^) and narrow LoAs, despite the fact that the sine-wave model [10] was originally validated for horizontal running. The fact that agreement remained excellent at this gradient suggests that the Stryd algorithm may account for gradient-induced mechanical changes, though the specific mechanism cannot be determined from available data. The slightly wider LoAs at +12% versus 0% grade (±0.61 vs. ±0.59 kN·m^−1^) are consistent with expected additional noise on inclines and remain within acceptable ranges. The marginally lower ICC at +12% (0.959 vs. 0.970) most plausibly reflects the mechanical constraints imposed by uphill running, where mean flight time was substantially shorter (56.7 ± 29.7 ms vs. 96.0 ± 19.4 ms), with some individuals approaching near-zero aerial phase. The Morin [10] sine-wave model assumes a distinct flight phase for estimating peak ground reaction force (Equation (1)); when flight time approaches zero, the model’s assumptions are more likely to be violated, which may introduce additional estimation error. Although this did not prevent excellent overall agreement, future studies should consider minimum flight-time thresholds when applying the sine-wave method at steep gradients where participants adopt a walking or bounding pattern.

While cadence, stride length, contact time, and power have been progressively validated [1,2,6], k_leg_ has remained the least characterized Stryd output; thus, this study closes a key evidence gap. In the context of the broader spring–mass model literature [8,9], the negligible systematic bias observed here suggests that the Stryd algorithm captures the mechanical determinants of k_leg_ (namely contact time, flight time, speed, and leg length) with sufficient fidelity to reproduce Morin’s sine-wave estimates [10]. This is noteworthy given that k_leg_ is strongly modulated by running speed and body mass [8], both of which vary across participants. The reliability of Stryd-derived contact time and speed—previously confirmed by Berzosa et al. [7] and Cartón-Llorente et al. [2] across terrain conditions—likely underpins the high agreement observed for k_leg_. Furthermore, Jaén-Carrillo et al. [11] recently demonstrated that k_leg_ derived from Stryd remained stable across 180 min of submaximal running and repeated uphill time trials, which contextualizes the present validation within a durability framework relevant to trail running performance. Future work should extend this validation to downhill and outdoor trail conditions. Level-2 and Level-3 validation represent the next logical steps.

These findings have direct practical implications. Stryd k_leg_ values showed negligible systematic bias and low random error relative to Morin-derived estimates, supporting their use for continuous field monitoring without laboratory-based calculations, under the conditions studied here. This is particularly relevant for trail running and mountain racing, where uphill running constitutes a substantial portion of total load and prior validation of Stryd on inclined terrain was lacking. Given that k_leg_ is associated with running economy, fatigue-induced gait alterations, and musculoskeletal injury risk [8,11], continuous Stryd k_leg_ monitoring may enable real-time detection of stiffness drift during prolonged efforts, with potential applications in pacing strategy and fatigue management [13]. Regarding the practical acceptability of the observed error, an MAPE < 3% is substantially below the 5% threshold recommended by Dhahbi & Chamari [5] and well within the range of day-to-day biological variability typically reported for k_leg_ in trained runners [8], making it unlikely to affect training monitoring decisions such as fatigue detection or injury early warning. In the broader wearable landscape, similar validation studies have assessed k_leg_ from devices such as Garmin [7] and RunScribe [2]; the Stryd MAPE values reported here are comparable to or lower than those reported for these devices, suggesting competitive measurement performance among commercially available foot pods.

Several limitations should be acknowledged. First, the sample consisted entirely of highly trained trail runners; it remains unknown whether the present findings generalize to recreational runners, beginners, or athletes from other disciplines with different biomechanical profiles. Second, validity was assessed only at a +12% uphill gradient and at 0% (level); steeper slopes or downhill running—common in trail and mountain racing—were not evaluated. Third, Morin’s sine-wave method, rather than a force platform, served as the reference standard; although this method has been independently validated against force platforms [10], it does not provide the same level of criterion validity as direct ground reaction force measurement. Fourth, leg length was estimated using a population-based anthropometric ratio (L = 0.53 × height) rather than measured directly, which may introduce individual-level error, particularly in athletes with atypical limb proportions. Future studies should address these limitations by including broader participant samples, a wider range of gradients, and direct leg length measurement.

## 5. Conclusions

This study provides Level-1 analytical validation of the Stryd leg stiffness metric against the Morin (2005) sine-wave reference method. Stryd k_leg_ demonstrated excellent agreement under both level running (ICC = 0.970, MAPE = 2.6%) and uphill treadmill running at +12% grade (ICC = 0.959, MAPE = 2.4%), with negligible mean bias and no statistically significant systematic error in either condition. Given the growing use of Stryd in running and trail running research and the scarcity of prior evidence on k_leg_ validity, particularly on inclined terrain, these results fill a critical evidentiary gap. Stryd k_leg_ satisfies all Level-1 validity thresholds and may be used with confidence as a continuous wearable field measure of leg spring stiffness.

## Figures and Tables

**Table 1:** Descriptive statistics and leg stiffness by condition (mean ± SD).

Variable	Uphill Running (+12%)	Level Running (0%)
Speed (m·s^−1^)	2.59 ± 0.34	3.10 ± 0.41
Contact time (ms)	290.5 ± 29.0	262.2 ± 26.1
Flight time (ms)	56.7 ± 29.7	96.0 ± 19.4
k_Stryd (kN·m^−1^)	9.33 ± 1.09	9.09 ± 1.22
k_Morin (kN·m^−1^)	9.35 ± 1.05	9.14 ± 1.22
Bias: Stryd—Morin	−0.02 ± 0.31	−0.04 ± 0.30
MAPE	2.5%	2.6%

k_Stryd = Stryd-reported leg spring stiffness; k_Morin = leg spring stiffness estimated via the sine-wave method; Bias = k_Stryd—k_Morin; MAPE = mean absolute percentage error.

**Table 2:** Level-1 analytical validation statistics.

Statistic (Threshold)	Uphill Running (+12%)	Level Running (0%)
ICC(2,1) [threshold: >0.7]	0.959	0.970
95% CI for ICC(2,1)	[0.903, 0.982]	[0.929, 0.987]
Pearson r (R^2^)	0.958 (0.919)	0.969 (0.940)
Paired *t*/*p*	0.25/0.803	0.68/0.505
Bland–Altman Bias (kN·m^−1^)	−0.016	−0.043
LoA (kN·m^−1^)	[−0.595, +0.628]	[−0.549, +0.634]
MAPE	2.44%	2.59%

ICC(2,1) = intraclass correlation coefficient, two-way random effects model, absolute agreement, single measures; 95% CI = 95% confidence interval computed via the F-distribution method; R^2^ = coefficient of determination; paired *t*/*p* = paired *t*-test statistic and *p*-value comparing k_Stryd and k_Morin; LoA = 95% limit of agreement (mean bias ± 1.96·SD); MAPE = mean absolute percentage error.

## Data Availability

The data presented in this study are available on request from the corresponding author. The data are not publicly available due to authors’ preferences.

## Abbreviations

The following abbreviations are used in this manuscript:

Units of measurements	
kN·m^−1^	Kilonewtons per meter
m·s^−1^	Meters per second
ms	Milliseconds
kg	Kilograms
m	Meters
%	Percentage
mmol·L^−1^	Millimoles per liter
Biomechanics and running variables	
k_leg_	Leg spring stiffness
CT	Contact time
FT	Flight time
v	Running speed
L	Leg length
F̂_max_	Modeled peak ground reaction force
Δŷ_c_	Peak leg spring compression
g	Acceleration due to gravity (9.81 m·s^−2^)
Statistics	
ICC	Intraclass correlation coefficient
MAPE	Mean absolute percentage error
LoA	Limit of agreement
95% CI	95% confidence interval
r	Pearson correlation coefficient
R^2^	Coefficient of determination
SD	Standard deviation
BIAS	Systematic error of mean difference
